# The Work of the Holy Ghost

**Published:** 1887-05-28

**Authors:** 


### Words of Consolation.

[Communications for this column should be addressed, "Chaplain," care of the Editor of The Hospital, who will gratefully welcome any suggestions or inquiries from matrons, nurses, and the staff generally.]

### XXXV.—The Work of the Holy Ghost.

Dost thou believe in the Holy Ghost? Numbers of Christians who would answer "yes" to this question, would be sorely puzzled to explain what practical effect such a belief has ever had upon their lives. So far as any realisation of God being within them, moving them, striving with them, is concerned, they might answer more truly with the disciples of Ephesus, "We have not so much as heard whether there be any Holy Ghost" (Acts xix, 2). Now the moral of what has been written so far upon this subject is to give the Holy Ghost credit for His work in us. (1) Convictions of sin. We have had them. They have been unwelcome. Nevertheless, they should be accepted with gratitude, with reverence, with humility; for they are the voice of God pleading for repentance. (2) Convictions of righteousness. The drawing of the soul to Christ. The leaning upon Him for salvation. The desire to become righteous; then the assurance of holiness being possible, even in a world of sin. Such impressions carry yet more weight if you reflect who it is that produces them. (3) Convictions of judgment. The day very present to the mind. The certainty of the second Advent. "Behold, He cometh with clouds, and every eye shall see Him; and they also which pierced Him" (Rev. i, 7). Accept these thoughts whenever they enter the mind, and you will then be found living in the spirit of God's holy fear.

But, beyond the acceptance of the most palpable convictions, there is in the lives of those who have become obedient to the Holy Ghost a constantly abiding habit of responding to His inspirations. These will be more fully treated when we come to speak of His guidance. Meanwhile we should observe that the disciple who has learnt to value convictions of the first magnitude (we call them so because obedience or disobedience to these is a matter of life or death) soon acquires a habit of scanning and rating at their proper value the multitude of thoughts, impulses, inclinations, wishes, likes, and dislikes, which daily traverse the mind. Out of these a certain number are discerned which bear the superscription "I ought," or "I ought not"; and these last in turn, however trivial, apparently, the circumstances which have influenced the mind, are also recognised as convictions, and obeyed accordingly. Indeed, belief in the Holy Ghost is never so practical as in minds disciplined to watching for and discerning the pressure which He puts upon them in little matters, deemed by most people scarcely worth a moment's consideration. Strong, deep, sometimes very terrible and distressing, convictions are essential for the conversion of certain souls. But once these have been given and obeyed, the progress of sanctification will chiefly depend upon whether the same Divine Person is recognised equally in His more gentle pleadings as "the wind bloweth where it listeth."

(*To be continued.*)

